# Notes and News

**Published:** 1892-06-18

**Authors:** 


### Notes and News.

COLLECTED AND COMMUNICATED.

The Children's Floral Fête at the Royal Botanic Gardens, Regent's Park, to be held on Wednesday, June 22nd, is a form of entertainment which offers attractions to many classes of the community, and especially to those who are unable to take part in other festive scenes. To those going through the tedious stages of convalescence, so varied and pretty a scene combined with the restful beauty of the gardens, should especially commend itself, and we can imagine few unable to appreciate so unique a spectacle. Prizes are offered for children's flower-dressed carriages, designs and groups of flowers, etc., which calls forth a display of much taste and ingenuity. Tickets are to be obtained through fellows of the Royal Botanic Society.

The English have the reputation all the world over of being a very self-satisfied nation. Perhaps the very appreciation of our own good qualities stimulates us as a nation to cultivate them. Undoubtedly English benevolence is not a virtue kept for home use only Wherever we travel fresh instances meet us to assure us of this. Not long ago we were visiting Dinard, a watering place in Brittany, and were much pleased and surprised to find that the little English colony there had established a small hospital during the past year not for such of their fellow-countrymen who chose to, avail themselves of its benefits, but for the succour of the poor of the native population, who, having long distances to go for hospital aid, are really, on account of their poverty, in even greater need of assistance than their English brethren. The happy idea emanated from Mr. Arthur Gardiner, sometime resident in Dinard, and a small committee assist him in the management. The nursing and entire work of the hospital is conducted by the excellent Sisters Trinitaire de Valence. Miss Charlotte Monteith, the able and energetic Secretary, contributes much towards the well-being of the little institution, and we hope that during the coming season her efforts will be encouraged by increasing contributions on the part of the many English visitors. There is a ladies' club in Dinard, and we hope we shall hear that the management has been induced to lend its aid by giving a series of entertainments for the benefit of the hospital

The Lambeth Board of Guardians are contemplating purchasing the convalescent home at Margate, where inmates of the Lambeth Workhouse have been usually received, as the establishment as now existing is about to be closed. The home contains from 200 to 300 beds.

The fund which is being raised for the purpose of building a new wing (to be called the Clarence Memorial Wing) at St. Mary's Hospital, received substantial aid on the 9th inst. from the special matinée given, by Mrs. Tree's kind permission, at the Haymarket Theatre. We were pleased to see several nurses amongst the large audience, which assembled to enjoy a variety of performances, each perfect in its kind. The generosity of "players" is a well-recognised fact, and it is never more lavishly displayed than when the sick and suffering require assistance; then, indeed, the greatest talent in London cheerfully lends itself to the cause. We fancy that all who bought tickets for this matinée must have realised that any "charity" in so doing was very considerably reduced by the large discount they received in the form of real personal enjoyment.

The St. John's Foundation School for Sons of Poor Clergy, which is about to hold its festival dinner, was founded just forty years ago, when some gentlemen started a scheme for providing a free education for the sons of a few poor clergymen. At first this was done on a very small scale; some eight or ten boys were boarded and educated in the house of the curate of St. Mark's Church, St. John's Wood, but it was found that the need for such a school was so urgent that a different arrangement soon had to be made. Little by little the school has grown, until last year there were 217 boys on the foundation, of whom 150 were on the original foundation, and 67 supplementary foundationers, who pay thirty guineas a year for board and education. During the year an additional block of buildings, to accommodate 60 boys, was opened, and is now nearly full. It is to be doubted whether the public realise how many clergymen of the Church of England, while doing good work in their parishes, are hardly able to supply their families with the necessaries of life. It is greatly to the credit of these poorer clergy that they generally bear their burdens in silence, not considering it consistent with their position as gentlemen to make known their wants. The one assistance, however, they feel they can accept is the education of their children. A large number cannot possibly afford to send their boys to school; this difficulty is becoming greater, for, while the incomes of the clergy are diminishing with the tithe, the cost of education is increasing. The Committee of the school are anxious to add to their numbers. They have room for and plans for three additional blocks, each of which would cost about £7,000, and accommodate 60 boys; but before they contract for another block they must build a dining-hall and kitchen, as the present dining-room will not accommodate any larger number of boys. The cost of these four buildings would not be less than £30,000. The Chairman is sanguine that, before many years are over, these buildings will be provided, but in the meantime the collection annually of sufficient funds to support the present establishment is a cause of no small anxiety, and he most earnestly appeals for assistance to continue and extend the school.

